# Supplementary material for: The genome sequence of a leaf-roller moth, Argyroploce noricana (Herrich-Schaffer, 1851) (Lepidoptera: Tortricidae)
Source: Wellcome Open Res. 2026 Sep 14;11:637. [Version 1] doi: 10.12688/wellcomeopenres.27791.1 (PMC13654068; doi:10.12688/wellcomeopenres.27791.1)
Supplement: Peer review history

# Reviewer report: Reviewer response for version 1

**DOI:** 10.21956/wellcomeopenres.30584.r170586

**Published:** 2026-10-20

**Authors:** Marta Vila

**Affiliations:** 1 Universidade da Coruna, Galicia, Spain

This Genome Note by Mutanen *et al.* presents a haplotype-resolved, chromosome-level assembly of *Argyroploce noricana*, an Arctic–alpine leaf-roller moth listed as Endangered in Finland, generated within Project Psyche from PacBio HiFi and Hi-C/Illumina data. The assembly is of excellent quality. Contig N50 is 20.3 Mb, 99.7% of the sequence is assigned to 26 chromosomal pseudomolecules, QV exceeds 65, and BUSCO completeness is 98.8%. The resource will be valuable for resolving the unstable generic classification of Olethreutini and for comparative studies of chromosome evolution in Lepidoptera. The text is clear and the species background is informative. I have read the two previous reports and largely agree with them. Below I indicate which points I consider most important, and add several additional observations.

**Comments on previous reports**

 I strongly support Dr Kim’s suggestion (point 1) to extract the COI barcode region from the assembled mitogenome and compare it with BIN BOLD:AAD8744 and with *A. aquilonana*. The specimen was collected at Saana, Kilpisjärvi, in the only region where the two species are sympatric, and identification is reported as morphological only. This check is quick and would make the identity of the reference specimen verifiable.

 I also strongly support Dr Lantz’s point 1 on voucher material. Only the head (Hi-C) and part of the abdomen (HiFi) were used, so the remainder of the specimen should exist. Its repository and catalogue number should be stated in the text, and the BioSample “specimen voucher” field (currently “not provided”) should be updated.

 Regarding the blob plot (Figure 7), the legend contains only two categories, “Arthropoda” and “no-hit”. The small off-cluster scaffolds therefore appear to be unassigned rather than assigned to contaminant taxa. A brief statement in the caption clarifying this, and confirming that ASCC flagged no contaminants, would address both reviewers’ comments. The isolated point at roughly 18% GC with very high coverage is presumably the mitochondrial genome. Identifying it as such would also explain the discrepancy between 101 scaffolds in Figure 6 and 100 in Table 2 (Dr Kim, point 5).

 On scale (Dr Kim, point 2), the voucher photograph appears to be taken on millimetre graph paper. Stating this in the caption, together with the wingspan already given in the Background, would suffice.

**Additional comments**

1. **Z chromosome and karyotype evolution.** The Z chromosome is by far the largest element in the assembly (173.4 Mb, about 22% of the genome, more than three times the largest autosome). According to Table 3 and Figure 4, it comprises MZ together with M17, M2, M20 and M22. Two further autosomal fusions (M13+M5, M18+M26) reduce the karyotype to n = 26 from the ancestral 31. I think this is the most interesting biological feature of the genome. I suggest one or two sentences addressing:
  - whether the Z–M17 fusion and the additional fusions involving M2, M20 and M22 are shared with other sequenced Olethreutini or Tortricidae, or appear to be lineage-specific;
  - any information (if available) about sex determination in this genus/family, given the fact that the sequenced specimen is male so that there is no  info about the W chromosome.
2. **Taxonomic assignment of a chromosome-level scaffold.** The Figure 7 legend reports 25 scaffolds (782 Mb) assigned to Arthropoda and 76 “no-hit” scaffolds totalling 17.4 Mb with an N50 of about 15 Mb. Since the assembly contains 26 chromosomes and unplaced sequence accounts for only about 0.3% of the assembly, this suggests that one chromosome-level scaffold of roughly 15 Mb received no taxonomic hit. Could it be chromosome 22 (OZ275226.1, 15.04 Mb)?
3. **Chromosome 9.** Chromosome 9 has the highest GC content in the assembly (39%) and shows elevated Hi-C contacts with most other chromosomes in Figure 3. It is also where the contig order and orientation are uncertain. Could the authors comment on whether this reflects a repeat-rich region, for example the rDNA array? The high-coverage unplaced scaffolds at 45–48% GC (Dr Kim, point 6) may be related.
4. **Hi-C read counts.** The text states that Hi-C sequencing produced “465.07 million reads”, whereas Table 1 gives 465.07 million read pairs. The base count (140.45 Gb) is consistent with read pairs of 2 × 150 bp. The same sentence states that Hi-C data were used to scaffold “the haplotype 1 assembly”, although both haplotypes were scaffolded. I recommend to clarify both issues.
5. **Table 4.**
  - Only for the downloaded pdf: The “#” symbols in the Benchmark column (“# 40”, “# 90%”) are typesetting error for “≥”. Check with the WOR production office.
  - The per-haplotype *k*-mer completeness values (84.2%) are below the stated “>90%” benchmark, and only the combined value passes. This is expected for a phased diploid assembly with 0.67% heterozygosity, but a note to that effect would avoid confusion.
6. **Background.**
  - The authority is spelled “Herrich-Schaffer” in the title and “Herrich-Schäffer” in the text; please harmonise.
  - I suggest to rephrase the statement that *Dryas octopetala* as food plant “remains unverified” (given the the subsequent report of successful laboratory rearing on *D. octopetala* (Huemer, 1988)). Maybe just indicating that larvae accept *Dryas* in captivity but the host plant has not been confirmed in the field.
7. **Conservation status and permits.** Please provide a reference for the Endangered status in Finland (the Finnish Red List assessment). Given that the species is Endangered there, a brief statement on collecting permits (if needed) would also be appropriate.

In summary, this is a high-quality genome resource. Most of the points above are minor and easily addressed. Congratulations!

 I used an AI assistant (Claude, Anthropic) to help draft and organise this report. I have checked all points against the manuscript, and the assessment and conclusions are my own.

Are the datasets clearly presented in a usable and accessible format, and the assembly and annotation available in an appropriate subject-specific repository?

Yes

Are sufficient details of the sequencing and extraction, software used, and materials provided to allow replication by others?

Yes

Are the rationale for sequencing the genome and the species significance clearly described?

Yes

Are the protocols appropriate and is the work technically sound?

Yes

Reviewer Expertise:

Lepidoptera genomics

I confirm that I have read this submission and believe that I have an appropriate level of expertise to confirm that it is of an acceptable scientific standard.

# Reviewer report: Reviewer response for version 1

**DOI:** 10.21956/wellcomeopenres.30584.r172031

**Published:** 2026-10-15

**Authors:** Henrik Lantz

**Affiliations:** 1 Uppsala University, Uppsala, Sweden

This manuscript presents the genome sequence of the leaf-roller moth, Argyroploce noricana, assembled as a part of project Psyche. The species has a northern distribution in Europe and is originally described from the alps. The specimen here used was collected in Finland and is considered endangered there. The species is closely associated with Dryas octopetala, a plant species common in for example Scandinavia, associated with calcareous high altitude ground.

 The text is in general well written, and I appreciate the background of the species given. The assembled genome is of very high quality and can most definitely contribute to the study of the debated classification of the genus. All assembly metrics easily meet the Earth Biogenome Project criteria, with very high numbers in kmer completeness, BUSCO, and amount of assembly assigned to chromosomes.

 Methods follow a high international standard but the description of them lack details, which leads to some problems regarding reproducibility (see below).

 I have some comments, with more major issues at the top.

 1. It is unclear what has been done to preserve a voucher specimen. Figure 1, named "Voucher photograph" presents a photo of the specimen used in the study. Does this mean that the photo constitutes the voucher? Only part of the abdomen of the specimen was used, which means there should be material left to preserve as a voucher, which would in turn facilite later affirmation of the identity of the specimen. Looking in ENA Biosample, Specimen Voucher is given as "not provided", and this is troublesome. Especially for reference genomes, which might serve as the single example of a full genomic sequence of this species for quite some time, material (or in worst case a photo) must be preserved. It might very well be that there is voucher material preserved in a Natural History Museum or that the photo constitutes the voucher, but this should then clearly be spelled out.

 2. I have some comments regarding reproducibility, which is somewhat lacking. Tools are usually clearly described, including version of tool used, but very little is written regarding settings used for the tools. Settings can have a big influence on the results. Databases used (UniProt and NCBI nt) are also not described with version or date. It is also unclear which pipelines were used. A link is provided to a page with ToL-pipelines, and one can guess that the genome assembly pipeline has been used, but this is not stated. As the text is written now, it is not possible to reproduce the results of the study. A link to a GitHib repo with scripts used would solve these issues.

 3. I would prefer to see figures 3 (HiC contact map) and 7 (BlobToolKit blob plot) somewhat better described. The contact map shows connections between some scaffolds, most likely just shared repeats, but it would be good to have that described. The blob plot shows some contaminations remaining. It would be helpful to have that mentioned and why it might or might not be a problem for downstream analyses.

 To summarize, the here presented genome constitutes a very valuable and high quality resource. If some more information could be given regarding voucher material and tools used, the manuscript would be greatly improved.

Are the datasets clearly presented in a usable and accessible format, and the assembly and annotation available in an appropriate subject-specific repository?

Partly

Are sufficient details of the sequencing and extraction, software used, and materials provided to allow replication by others?

Partly

Are the rationale for sequencing the genome and the species significance clearly described?

Yes

Are the protocols appropriate and is the work technically sound?

Yes

Reviewer Expertise:

I have a long background in systematics, taxonomy, genome assembly, and genome annotation. I lack knowledge of the species group here studied, although I feel that does not impede my ability to review the manuscript. I only have general knowledge of the laboratory procedures described, and find it somewhat hard to comment on those.

I confirm that I have read this submission and believe that I have an appropriate level of expertise to confirm that it is of an acceptable scientific standard, however I have significant reservations, as outlined above.

# Reviewer report: Reviewer response for version 1

**DOI:** 10.21956/wellcomeopenres.30584.r172027

**Published:** 2026-10-10

**Authors:** Jinmu Kim

**Affiliations:** 1 University of Illinois at Urbana-Champaign, Urbana, Illinois, USA

**Article assessment**

 This Genome Note by Mutanen M. et al. reports a haplotype-resolved, chromosome-level genome assembly of a male Argyroploce noricana, an Arctic–alpine leaf-roller moth that is listed as endangered in Finland, generated from HiFi and Hi-C data. Overall, the findings provide a high-quality genomics resource. However, a few minor comments should be addressed before final indexing.

**Detail comments**

 1. The background states that A. noricana and the closely related A. aquilonana co-occur in the Scandinavian mountains, which is the only region where their ranges overlap, and the sequenced specimen was collected in exactly this region (Saana, Kilpisjärvi). Identification is reported as morphological only, and since the mtDNA has been assembled, the authors should extract the COI barcode region and report its similarity to BIN (BOLD: AAD8744) and to A. aquilonana barcodes. This additional analysis would make the identification of the reference specimen verifiable.

 2. Figure 1: What is the size of the specimen? Include a scale bar or indicate the length (mm, cm, etc.).

 3. Figure 3: The last 3 chromosomes are labeled only with dots on the top axis, so please label them. The uncertain regions on the chr 9 should be marked on the contact map with asterisks or other symbols and described in the caption, with a corresponding footnote in Table 3.

 4. Figure 5: The legend lists a red “hap1-only” curve; however, it is not visible because it is completely overlapped by the blue “hap2-only” curve. Use transparency or different line types or state in the caption that the two curves overlap.

 5. Figure 6: The snail plot reports 101 scaffolds, whereas Table 2 reports 100 scaffolds after excluding organelle sequences. The caption should clarify that the snail plot includes mitochondrial contigs.

 6. Figure 7: Several small unplaced scaffolds indicate coverage of 100-1000X and GC of 45-48%, outside the autosomal cluster. Briefly state what these represent (for example, rDNA, satellite, or repeats) and confirm that ASCC did not identify any contaminant sequences.

 7. Table 5: Explain why three different MultiQC versions were used.

Are the datasets clearly presented in a usable and accessible format, and the assembly and annotation available in an appropriate subject-specific repository?

Yes

Are sufficient details of the sequencing and extraction, software used, and materials provided to allow replication by others?

Yes

Are the rationale for sequencing the genome and the species significance clearly described?

Yes

Are the protocols appropriate and is the work technically sound?

Yes

Reviewer Expertise:

Whole genome sequencing, Whole transcriptome analysis, Shotgun metagenomics, Metabarcoding, Marine genomics

I confirm that I have read this submission and believe that I have an appropriate level of expertise to confirm that it is of an acceptable scientific standard.

# Reviewer report: Reviewer response for version 1

**Published:** 2026-09-14

**Authors:** Automated Benchmarking

**Affiliations:** 1 Wellcome Sanger Institute, Hinxton, UK

As this article is a Genome Note, Automated Benchmarking has been used to determine whether the reported assembly passes three defined checks established by the Earth BioGenome Project: the raw and assembled data are publicly available, the Earth BioGenome Project three-part metric is met, and the BUSCO score passes the stated threshold. For Argyroploce noricana (ilArgNori1), all three checks passed. Data available: pass. ENA study PRJEB89138, raw-read accessions ERR14943086 and ERR14941502, and assembly accessions GCA_965607585.1 and GCA_965607555.1 are public. Earth BioGenome Project three-part metric: pass. Haplotype 1 is reported as 7.C.Q65, meeting the 6.C.Q40 reference standard. BUSCO: pass. Haplotype 1, which is proposed as the reference assembly for this species, has 98.8% complete BUSCOs (98.1% single-copy and 0.7% duplicated). BUSCO software version: 5.8.3; lineage dataset: lepidoptera_odb10. It meets the target of greater than 90% complete and single-copy BUSCOs. This decision uses values reported in the Genome Note and public accession records.

Reviewer Expertise:

NA

I confirm that I have read this submission and believe that I have an appropriate level of expertise to confirm that it is of an acceptable scientific standard.
